# Herpes simplex virus type 1 and Alzheimer’s disease: increasing evidence for a major role of the virus

**DOI:** 10.3389/fnagi.2014.00202

**Published:** 2014-08-11

**Authors:** Ruth F. Itzhaki

**Affiliations:** Faculty of Life Sciences, University of ManchesterManchester, Lancs, UK

**Keywords:** herpes simplex virua type 1, Alzheimer’s disease, apolipoprotein E, brain, reactivation, antivirals

## Abstract

Herpes simplex virus type 1 (HSV1), when present in brain of carriers of the type 4 allele of the apolipoprotein E gene (APOE), has been implicated as a major factor in Alzheimer’s disease (AD). It is proposed that virus is normally latent in many elderly brains but reactivates periodically (as in the peripheral nervous system) under certain conditions, for example stress, immunosuppression, and peripheral infection, causing cumulative damage and eventually development of AD. Diverse approaches have provided data that explicitly support, directly or indirectly, these concepts. Several have confirmed HSV1 DNA presence in human brains, and the HSV1-APOE-ε4 association in AD. Further, studies on HSV1-infected APOE-transgenic mice have shown that APOE-e4 animals display a greater potential for viral damage. Reactivated HSV1 can cause direct and inflammatory damage, probably involving increased formation of beta amyloid (Aβ) and of AD-like tau (P-tau)—changes found to occur in HSV1-infected cell cultures. Implicating HSV1 further in AD is the discovery that HSV1 DNA is specifically localized in amyloid plaques in AD. Other relevant, harmful effects of infection include the following: dynamic interactions between HSV1 and amyloid precursor protein (APP), which would affect both viral and APP transport; induction of toll-like receptors (TLRs) in HSV1-infected astrocyte cultures, which has been linked to the likely effects of reactivation of the virus in brain. Several epidemiological studies have shown, using serological data, an association between systemic infections and cognitive decline, with HSV1 particularly implicated. Genetic studies too have linked various pathways in AD with those occurring on HSV1 infection. In relation to the potential usage of antivirals to treat AD patients, acyclovir (ACV) is effective in reducing HSV1-induced AD-like changes in cell cultures, and valacyclovir, the bioactive form of ACV, might be most effective if combined with an antiviral that acts by a different mechanism, such as intravenous immunoglobulin (IVIG).

## Introduction

The concept of a viral role in Alzheimer’s disease (AD), specifically of herpes simplex virus type 1 (HSV1), was first proposed several decades ago (Ball, 1982; Gannicliffe et al., 1986). Legitimizing the concept clearly depended on a positive answer to a number of test questions, the first of which was whether or not HSV1 is ever present in human brain. The subsequent discovery that HSV1 DNA resides in a high proportion of brains of elderly people in latent form (Jamieson et al., 1991)—both normals and AD patients—immediately made the concept more credible, but raised associated questions such as whether or not the virus is ever active in brain or is merely a passive resident there; whether on its own it is a causative factor in AD or it acts thus only with another factor, perhaps genetic; if active, what causes its activity; whether there is any link with the characteristic abnormal features of AD brains or their components, and whether, if indeed implicated in AD, antiviral agents would be useful for treating the disease. These questions were posed in a previous review (Wozniak and Itzhaki, 2010)—and strong evidence was presented that permitted the answer to each question to be “yes” or, very likely to be “yes”. The present review briefly summarizes the earlier evidence, and provides an update, which is especially timely in view of the subsequent steady increase in number of relevant publications.

In contrast to the high frequency of HSV1 DNA in elderly brains, the viral DNA was found to be present in only a very small proportion of brains of young people and children (Wozniak et al., 2005, 2009). It was therefore suggested that HSV1 enters the brain in older age, as a consequence of the decline in the immune system with age. Subsequently it was found that the virus in brain of carriers of the APOE-e4 allele confers a strong risk factor for AD (Itzhaki et al., 1997), accounting for some 60% of cases. Further, evidence was obtained indicating that HSV1 reactivates in brain and causes a productive infection, perhaps recurrently (Wozniak et al., 2005). Active HSV1 infection causes severe damage and usually leads to cell death. It was therefore suggested that HSV1 might periodically reactivate in brain during episodes of stress, immunosuppression or inflammation, causing cumulative—though necessarily limited and localized) damage—which is greater in APOE-e4 carriers, leading eventually to the development of AD. Reactivation events have long been known to occur in the peripheral nervous system, where HSV1 resides lifelong in the trigeminal ganglia; they cause overt damage in the form of cold sores (herpes labialis) in some 20–40% of those infected, whereas others remain asymptomatic—an enigma solved by work in the author’s laboratory (Itzhaki et al., 1997, and see below), and subsequently confirmed by another (Koelle et al., 2010).

HSV1 effects are both direct—including subverting the cell’s machinery to produce viral instead of cellular components, and indirect, via inflammatory processes. The neuropathological processes leading to the disease—which is generally acknowledged to be multifactorial—are unknown. However, in the case of HSV1 acting with APOE-e4, they might occur via the virus’s known ability to cause accumulation of beta amyloid (Aβ), and AD-like tau (P-tau), the main components respectively of the characteristic amyloid plaques and neurofibrillary tangles (NFT) of AD brains (see Itzhaki and Wozniak, 2012). Several studies add support to the above scheme: firstly, APOE has been found to modulate severity of disease (or susceptibility to infection) in the case of several diseases of microbial cause, including two caused by HSV1 and herpes simplex virus type 2 (HSV2)—namely, herpes labialis (Itzhaki et al., 1997) and genital herpes (Jayasuriya et al., 2008). For both of these, APOE-e4 is a risk factor, paralleling its proposed role as a risk for AD in the CNS; secondly, HSV1 DNA is located very specifically within the characteristic amyloid plaques in AD brains (Wozniak et al., 2009).

Reactivation of HSV1 in brain obviously does not lead to overt herpes simplex encephalitis (HSE), as that outcome would be probably be fatal unless diagnosed rapidly and treated immediately with antivirals. Therefore reactivation events must presumably be very localized or mild; in fact such limited and asymptomatic reactivations have been described in a number of cases of recurrent HSE (see below).

Consistent with the concept of a viral role in AD, at least two other viruses, measles and human immunodeficiency virus (HIV), can cause changes in brain similar to those characteristic of AD. Measles virus, years after the initial infection, can lead to a rare but very severe disease, subacute sclerosing panencephalitis, in which NFT appear in brain (Wisniewski et al., 1979), and HIV infection leads to the formation of AD-like tau (Anthony et al., 2006). Also, postencephalitic parkinsonism, which is thought to be caused by a virus, is characterized by NFT (Buée-Scherrer et al., 1997).

In the last 23 years, many studies have been published—some 37 of these by groups other than the author’s—using diverse approaches and yielding diverse types of information, all of which explicitly support, or are consistent with, the proposed role for HSV1 in AD. In contrast, there have been very few publications describing contradictory data. Both sets of studies will be described below, though briefly in the case of those that were cited in two previous reviews (Wozniak and Itzhaki, 2010; Itzhaki and Wozniak, 2012), but it is worth mentioning that despite the preponderance of supportive data, the concept of a viral role in the disease is still regarded by some as controversial. This is probably because certain aspects relating to virus “behavior”—or indeed of microbial behavior—are either not understood, not considered, or not even recognized, by some researchers in the field. Indeed, there is a long history of resistance to the concept of microbial involvement in certain chronic diseases, even when incontrovertible experimental evidence had been obtained (as, for example, when Peyton Rous discovered a cancer-producing virus (Dulbecco, 1976)).

The first of the two aspects of microbial action that needs to be recognized is that microbes can cause not only acute infections—acting as hit-and-run agents—flying in, causing damage, and then flying out of the body: they can cause chronic diseases too. In fact certain microbes can remain in the body lifelong, mainly in a dormant state but occasionally or eventually becoming activated, and they then cause damage, sometimes severe or even fatal, to their host. The second aspect is that response to a microbe—susceptibility to infection, and/or the extent of damage caused if infection ensues—can differ from person to person, determined by genetic factors or immune responses. Thus, the outcome can range from uninfected, to asymptomatic infection, to severe or fatal illness, with the likelihood that an asymptomatic person would be classed as a “control”, despite harboring the microbe. Indeed, in some diseases, the proportions of those infected in the patient and control groups are very similar. In other words, “infect” does not necessarily mean “affect”. Some of those researchers who object to a viral role invoke the first of Koch’s postulates—that the microorganism must be found in abundance in all organisms suffering from the disease, but not be found in healthy organisms—as an argument against these concepts. However, Koch himself realized that this postulate was invalidated by the fact that some microbes can cause asymptomatic infection. In any case, if the postulates are applied to viruses, they need to be modified in several respects, as viruses are not independent organisms but instead multiply only within cells.

This review deals not only with updates on topics that were covered in the author’s most recent review, but also with topics not covered previously, namely, the possibility that cytomegalovirus (CMV) rather than HSV1 is a cause of AD; epidemiological studies on HSV/HSV1-specific humoral responses; genetic studies; evidence of reactivation of HSV1 in the CNS; effects of infection of mouse brains on Aβ and P-tau; studies on HSV1-infected cell cultures; and the case for antiviral treatment for AD patients.

## Is CMV rather than HSV1 a cause of AD?

The possibility that CMV, another member of the herpes family of viruses, rather than HSV1 is a major factor in AD has been considered in a number of publications. This is based on the many studies that have implicated the virus in immunosenescence, although it should be said that its role in the latter is unexplained, and many aspects are controversial. However, what is presumably the most obvious requirement—the presence of CMV in brain of AD patients—has not been established. In contrast, HSV1 DNA has been found in human brain by seven independent groups, including the author’s, the latter using three different methods: solution and *in situ* PCR, and immunological (Of the sole contradictory studies, two detected HSV1 DNA in only a very small percentage of subjects, and the third showed only a trend for HSV1-APOE-ε4 association conferring a risk of AD; these and the HSV1-positive findings were reviewed by Wozniak and Itzhaki, 2010). A further general point should be considered (Itzhaki et al., 1997)—that brains cannot be described truly as virus-negative when based on analyses of necessarily small specimens of the 1.5 kg organ.

The first study to seek CMV (Taylor and Crow, 1986) used the pre-PCR technique of DNA hybridisation, at a stated detection limit of one viral genome per 50 cells. However, neither CMV nor HSV1 was found in normal or AD brains. The only other study, in the author’s laboratory, sought CMV DNA by PCR, and detected it in 36% of AD brains and 35% of age-matched controls (Lin et al., 2002a). However, it was stressed that the value might be artefactually high, as no account could be taken of virus residing in lymphocytes within blood vessels rather than in brain cells (Interestingly, CMV DNA was detected in a much higher proportion—93%—of brains from vascular dementia patients (Lin et al., 2002b)). In fact although CMV can cause encephalitis, the latter is extremely rare in the immunocompetent, occurring only in those with severe T-cell-mediated immune deficiency.

The other approach to investigating the possible role of CMV in AD has involved examining serum antibody titres to the virus, as well as to other viruses and bacteria, and seeking to relate the titres to the occurrence of AD and/or to changes in cognition. Again, the most comprehensive early study (Renvoize et al., 1987) gave negative results for adenovirus, chlamydia Group B, *Coxiella burnettii*, HSV, influenza A, influenza B, measles and *Mycoplasma pneumoniae*), as well as CMV, in that the titres were similar in AD patients and controls for each pathogen. However, a more recent study, with presumably greater sensitivity, by Strandberg et al. (2003) discovered that viral burden—seropositivity for HSVI, HSV2, or CMV—was associated with cognitive impairment among home-dwelling elderly individuals with vascular disease (mainly coronary heart disease), although no significant associations were observed between bacterial burden (*chlamydia pneumoniae* (Cpn) and *mycoplasma pneumoniae*) and cognition. Later, Strandberg et al. (2005) found maximally increased risk of cognitive impairment, in elderly home-dwellers, in association with a combination of three factors: seropositivity for herpesviridae, presence of APOE-ε4, and low education, the data indicating that not only APOE-ε4, but also the epsilon3 haplotype, is a risk factor for dementia.

Katan et al. (2013) similarly investigated infectious burden (IB) in multiethnic elderly subjects with vascular risk, measuring IgG levels for *Helicobacter pylori*, HSV1 and HSV2 and IgA for Cpn, and cognition by Mini-Mental State Examination (MMSE), at enrollment and on annual telephone interview. Higher IB was found to be associated with worse cognition. The authors, like Strandberg et al., concluded that most of the effects of IB on cognition were mediated by viral rather than bacterial burden. However, they found no APOE effect on IB in respect to cognition. Another study, by Tarter et al. (2014), examined CMV and HSV1 seropositivity over a range of age groups in the general population. They found that HSV1 seropositivity in children was associated with impaired cognition, in the middle-aged with impaired reading and visuospatial processing and slower coding speed, and among older adults with immediate memory impairment. In contrast, CMV seropositivity was associated only with slower coding speed and impaired learning and recall in the middle-aged. They therefore suggested that HSV1 might have a life-course effect on cognition, while CMV effects might be restricted just to the middle-aged. As the age of primary infection with herpesviruses is affected by socio-economic factors—rising with increasing levels of the latter—the authors pointed out that these pathogens might contribute lifelong to “social disparities in cognition, educational attainment and social mobility”.

Westman et al. (2014) examined in AD patients and non-demented controls the functional capacity and cytokine release profile of peripheral blood mononuclear cells (PBMCs) in response to CMV and Aβ antigen challenge, and APOE genotype and systemic inflammatory biomarkers. Their data, and the evidence from other studies that HSV-1 resides in brain of AD patients, plus the epidemiological links to negative cognitive effects at all ages (Tarter et al., 2014), led them to propose that CMV deregulates inflammatory responses; it could thereby cause frequent or prolonged HSV-1 reactivation, increasing the risk of AD. An alternative mechanism was that CMV infection might directly affect AD pathogenesis by increasing local inflammatory response to various antigens, possibly including Aβ, increasing amyloid precursor protein (APP) production and formation of toxic amyloid protofibrils, leading to cognitive decline. In either case, CMV was posited as an inflammatory promoter in AD immunology. As the authors mention, this conclusion was consistent with the results of Stowe et al. (2012). The latter showed, in a very informative study of 1454 multiethnic subjects aged 25–91, that high antibody titres to HSV1—presumably reflecting high reactivation rates—were significantly associated with both CMV seropositivity and with high CMV antibody levels; also, increases in HSV1 antibody level with age occurred in CMV-seropositive people—just in those with low CMV antibody levels, since individuals with high levels already had high HSV1-antibody levels, but did not occur in people who were CMV-seronegative. Stowe et al. concluded that CMV can influence immune response to other pathogens, and might trigger the immune dysregulation involved in some age-related diseases, and they suggested specifically that HSV1 reactivation is associated with CMV and age.

Lurain et al. (2013) analyzed serum and cerebrospinal (CSF) fluid for associations between CMV infection and AD. They found that CMV-specific serum IgG antibody levels were associated with NFT of AD brain, as, also, was CSF interferon γ, which was detected only in CMV-seropositive patients; also, the percentage of senescent T cells was significantly higher in CMV-seropositive than in seronegative patients. Although the authors considered that CMV rather than HSV1 was implicated in AD (but see comment by Itzhaki and Klapper, 2014), their data were wholly consistent with the concept that inflammation caused by infections can affect the brain, resulting in reactivation of HSV1 resident in the CNS.

## Epidemiological studies on HSV/HSV1-specific humoral responses

Several studies have sought anti-HSV^1^ IgM as well as IgG in serum from AD patients, on the basis that the presence of IgM is associated with recent reactivation of HSV1, in contrast to IgG, which shows only that the person has been infected with HSV1. It should be added though that serum antibody levels reflect the response to the virus in the periphery; whether or not it reflects also the response to virus in the CNS is uncertain. However, it does seem likely that events that cause reactivation in the periphery, such as stress and immunosuppresssion, would cause reactivation also in the brain, but perhaps less frequently, and certainly in brain they might be much more difficult to detect.

The first study on anti-HSV IgM, by Letenneur et al. (2008), revealed that elderly subjects who were IgM-positive were far more likely to develop dementia within the following 14 years than were those who were IgG- but not IgM-positive, thus supporting the concept that reactivation of the virus leads eventually to dementia. Subsequently, Féart et al. (2011) found that high IgM levels, but not IgG levels, are associated with low plasma levels of Aβ1-40 and 1-42—which are considered to be biomarkers of the disease, in that their decrease might reflect accumulation of Aβ in brain cells (and hence a reduction in plasma level). Although the authors considered that virus reactivation might occur as a consequence of an initial accumulation of Aβ in brain rather than being a cause of accumulation, nonetheless they concluded that their data supported the involvement of virus reactivations leading to alterations in APP processing, and eventually to AD. No association of APOE-ε4 with IgM positivity was found in either study, which the authors tentatively attributed to the small number of people who were APOE-ε4 carriers and IgM-positive. Alternatively, this could perhaps be explained, as in other cases in which there apparently no APOE effect, if the latter determines severity of damage caused by reactivation rather than occurrence or frequency of reactivation (see e.g., Wozniak et al., 2005).

Lövheim et al. (2014) carried out a longitudinal prospective cohort study, investigating a possible relationship between HSV infection and AD by examining serum anti-HSV antibodies, and several neuropsychological, social and professional features. As the authors stress, the study was especially strong in that the design was prospective, the number of participants large, the follow-up duration lengthy, and the diagnostic evaluations numerous. Their first cohort of 1000 people was recruited in 1998–1990 and these were followed every 5 years thereafter. Other cohorts, each comprising around 1000 people, were recruited 5 years, 10 years and 15 years later, and in one case, participants were followed every successive 5 years. The ages of the subjects ranged from 35 years to 95 years. At all time points, a battery of tests was used to assess participants’ memory systems, problem-solving and decision-making ability, etc., and their health was examined in detail. The results were consistent with those of Letenneur et al. (2008) and Féart et al. (2011), in revealing that the presence of anti-HSV IgM antibodies—indicating HSV reactivation—but not the presence of IgG antibodies, almost doubled the risk of AD.

Another HSV antibody study, by Kobayashi et al. (2013), investigated the IgG avidity index (AI) in relation to cognitive function in AD and in amnestic mild cognitive impairment (aMCI). The avidity is defined as the strength with which IgG attaches to antigen, IgG avidity maturing with length of time following primary infection: thus, IgG produced within the first few months after primary infection exhibits low avidity, whereas IgG produced several months or years later exhibits high avidity; therefore the avidity can distinguish primary infection from past, long-lasting or recurrent infection. The authors took AI as an indicator of HSV1 reactivation, and scored patients’ MMSE and frontal assessment battery. They found that those with aMCI had a higher AI than healthy controls and, surprisingly, higher than AD patients, suggesting that more frequent reactivation occurs in the aMCI group. Also, the anti-HSV1 IgG AI and IgG antibody titre differed between the controls and the aMCI groups, indicating that HSV1 is involved in the change from a healthy state to the onset of aMCI. The apparently lower reactivation rate in AD might be explained by the occurrence of structural and functional changes in brain caused by HSV1 (or other agents/factors), at some time before the onset of confirmed AD.

An intriguing variant of HSV antibody studies, by Mancuso et al. (2014), examined serum anti-HSV1 IgG titres of patients with mild AD, and cortical gray matter volumes, measured by MRI. As expected, IgG levels were similar for patients and age-matched normal people. However, high antibody levels were significantly more frequent in the patients, and correlated positively with cortical bilateral temporal and orbito-frontal gray matter volumes. To find if these features were specific for HSV1, similar analyses were carried out for CMV antibodies. However, no such correlation was found, strengthening the case for HSV1 involvement. The authors commented that damage to the blood-brain barrier (BBB) might enhance immune cell entry, and suggested that HSV1-specific antibodies might play a protective role at early stages of AD by reducing HSV1 activity in brain regions where the BBB is disrupted.

## Genetic studies

Several genome wide association studies (GWAS) have examined genetic links between HSV1 and its host cells, generating complex papers with particularly imaginative titles! Licastro et al. (2011) reviewed recent data showing that a limited number of genes, when combined, are strongly associated with AD, even though the effect of any single gene or SNP was very weak (the OR values for AD in every case being less than 2, apart from that of APOE). They suggested that these genes might code for proteins that interact in various processes, thus leading to a synergistic effect on AD pathogenesis. Various possible mechanisms were considered, including environmental agents triggering certain genes, which might then affect other genes, thereby causing secondary effects via apoptosis, immune responses, etc. The authors’ main proposition was that microbes, in particular herpesviruses, might well be the link for all SNP that GWAS has shown to be associated with AD: thus, the first set of genes found were located close to the APOE locus, on chromosome 19; the second set were located on different chromosomes, and an earlier study (Porcellini et al., 2010) linked both sets, together with the PICALM gene, with susceptibility to herpes virus infection of the brain. A third set of five such genes gave further support to the virus infection hypothesis, through the genes potentially influencing one of the following processes: virus transport to the brain, and circulation within it, virus entry into neurons, regulation of defence mechanisms against the virus such as the host immune responses, cell sensitivity to apoptosis, or individual susceptibility to infection. They concluded from these data, as well as experimental work on HSV1 and AD by others, and studies on SNPs in other genes involved in various inflammatory responses—some of which have been associated with viral infections—that “viruses of the herpes family are among the most probable pathogen candidates (responsible for) CNS neurodegeneration in old age”.

Carter (2013) commented that HSV1 binds to many cell proteins, thereby modulating probably their expression, including many encoded by susceptibility genes for various neurological diseases, including AD. The interactome is estimated to be 1347 genes, in a currently estimated total protein-coding genes number of about 25000. The close association between viral interactomes and disease susceptibility genes suggests that there might be multiple gene/environment interactions which would allow pathogens to cause disease in genetically susceptible individuals. Surprisingly, the author stressed the point that the seroprevalence of HSV1 is much greater than that of the disease and so “has perhaps militated against a primary causal involvement”, even though he subsequently conceded that host and microbial genetics play a key role in determining outcome of infection. He commented also that even though viral detection, antiviral drugs and vaccination might affect the incidence of many diseases, host/pathogen protein mimicry suggests that the effects of treatments might be affected by autoimmune features.

## Evidence of reactivation of HSV1 in the human CNS

A major problem in studying the role of HSV1 in AD is the current lack of a method for detecting reactivation of the virus particularly if, as postulated, it occurs on a limited scale or in very localized regions of the CNS. Obviously, the gross damage caused during HSE is far more readily detectable—nowadays by seeking HSV1 DNA in the CSF, in which it remains for about a week after the start of the outbreak. However, there have been a number of case reports of HSE recurring some months or years after the initial episode, and it has been suggested that there might be fairly frequent occurrences of sub-clinical encephalitis which, because of their mildness, might not be diagnosed correctly (Klapper et al., 1984).

Interesting data were obtained by Peter and Sevall (2001) on analyzing 3200 CSF specimens, from subjects of a wide range of ages, sent to a reference laboratory for HSV testing. They found that over-all, 26 were positive for HSV1 and 36 for HSV2, but in the over-60 age group, the ratio of HSV-2–HSV-1 infection was low, namely, 3:13, and 11 of the 13 HSV1-positive subjects were female. In the over 70 s, comprising 1 male, 10 female, 10 were positive for HSV-1, with 1 female positive for HSV2. Over all, there were almost twice as many HSV-1 and HSV-2 infections detected in females as in males. They commented that their data display a particular bias for HSV1-CNS infection in females aged over 70 years—an intriguing finding, in view of the preponderance of females with AD.

A pre-PCR study on HSV1 in *post mortem* human brain specimens strongly suggested that HSV1 reactivates there under conditions such as immunosuppression (Saldanha et al., 1986). Brain from patients with acute leukaemia, who had been immunosuppressed as part of their treatment, was examined by *in situ* hybridisation using an ^3^H-HSV1 probe. Strong labeling, indicating the presence of HSV1 DNA, was revealed in frontal and temporal cortices of those who were HSV-seropositive but not in those who were HSV-seronegative or who had not been immunosuppressed. The incidental but salient point was made that in view of the subsequent mandatory usage of antivirals to treat HSE, such an investigation is unlikely ever to be carried out again.

Significantly, a review of multiple sclerosis patients treated with natalizumab, which hinders inflammatory cell migration into the CNS, revealed that between November 2004 and December 2012, 20 cases developed HSE (Fine et al., 2013), indicating the presence—and reactivation—of HSV1 in the patients’ brains. The authors commented that such treatment had in some cases led to the patients developing progressive multifocal leucoencephalopathy (caused by JC virus in brain, normally controlled by the immune system); however, other CNS opportunistic infections had rarely been reported, so they urged greater awareness of this risk.

## Effects of HSV1 infection of mouse brains relating to AD, including evidence of reactivation

Martin et al. (2014) examined the CNS and trigeminal ganglia of HSV1-infected mice for several markers of inflammation and neurodegeneration, with the aim of finding evidence of asymptomatic reactivation of HSV1. They detected viral ICP4 protein not only during the acute, symptomatic phase of infection at 7 days but also at 60 days post-infection, well after the start of the asymptomatic latent phase, thus indicating that reactivation was occurring. Up-regulation of Interferon α and β and toll-like receptors (TLRs) occurred concurrently, indicating neuroinflammatory action, consistent with their previous study showing that HSV1 activates TLRs 2 and 4 in mouse astrocyte cultures (Villalba et al., 2012), and that of Aravalli et al. (2005) in microglial cells. Phospho-tau and caspase-3-cleaved tau, which indicate early neurodegenerative processes, were up-regulated also. The authors concluded that their data support the hypothesis that HSV1in brain reactivates recurrently, thereby promoting neuroinflammation by triggering TLRs activation, thus conferring a risk of neurodegeneration.

The interaction of aging, HSV1 infection, gender and APOE in mouse brains was investigated by Guzman-Sanchez et al. (2012), who infected 2 month-old male and female wild-type and APOE-knock-out mice, and studied them over the following 16 months. Previous work by the same group (Burgos et al., 2003, 2006), had shown that APOE determines the viral load in the CNS during the acute and latent phases of infection, and was greater in APOE-ε4-transgenic than in APOE-e3 animals; also the load in female brains was greater than in males. Their 2012 study found that the viral load in brain was maximal during the acute phase, declined during adulthood and then increased with ageing, and that in wild-type female mice the load was 43 times greater than in apoE-K/O animals. Surprisingly, there were no detectable morphological or pathological differences between infected and mock-infected old animals. However, infection caused a memory deficit and reduced a metabolic measure of neuronal health. The authors commented that their results might explain the data indicating that the combination of HSV1 in brain and APOE-e4 carriage confers a strong risk of AD.

Sy et al. (2011) postulated that infections might promote the development of AD by exacerbating pre-existing neuroinflammation, hence accelerating cognitive decline. They induced inflammation in mouse brain by viral infection or lipopolysaccharide (the latter mimicking bacterial infection), to find if the inflammation modulated the AD-like features that develop in aged triple-transgenic (3xTg-AD) mice. A single dose of mouse hepatitis virus was used, thus causing an acute infection and a strong neuroinflammatory response. It was found that immune responses and mortality did not differ between the 3xTg-AD and non-Tg mice. No accumulation of Aβ occurred, but tau phosphorylation increased in the 3xTg-AD mice, though not in the non-Tg animals, and it increased also at 2 and 4 weeks post-injection, after the viral infection had been cleared, and when no short-term immune responses remained. Also, at the later stage, both groups of infected mice showed spatial memory impairments and demyelination in the spinal cord. Repeated treatment with LPS, which induced a sustained neuroinflammation, caused these changes too. The authors suggested that similar effects of infection might occur in human brain, thereby exacerbating tau pathological features, and leading to AD.

## Comparison of AD biomarkers and other AD proteins with proteins of known CNS infections

A novel study by Krut et al. (2013) compared CSF biomarker profiles of AD with various CNS infections, including HSE. Values for AD and HSE, in contrast to the other infections (Lyme neuroborreliosis, bacterial meningitis and HIV-associated dementia (HAD)) showed a markedly similar pattern in all but one respect: Aβ1-42 levels were lower in AD and HSE than in healthy controls, and those of total tau (t-tau) and hyper-phosphorylated tau (P-tau) were higher in AD and HSE than in the controls, but none of these values differed significantly between the AD and the HSE patients. Levels of soluble APP, however, were significantly lower in HSE than in AD. The authors therefore suggested that the mechanisms underlying the disturbed Aβ metabolism in the two disorders might differ, while the similarity of their P-tau and t-tau values might indicate that HSV1, as well as causing hyperphosphorylation of tau in cell cultures (see below), causes it also *in vivo*.

An interesting overlap of HAD proteins and AD proteins was discovered by Zhou et al. (2010), who analyzed total proteins from frontal cortices of HAD and HIV non-demented patients: 90% of the proteins they identified had been reported previously for several neurological diseases including AD (referred to by the authors as a “non-viral” disease for comparison with HIV, despite their citing several studies on AD viral aetiology!). Indeed, as they pointed out, their data might help to elucidate the involvement of any pathogen in “non-viral” neurodegeneration.

## Studies on HSV1-infected cell cultures

In the author’s two preceding reviews (Wozniak and Itzhaki, 2010; Itzhaki and Wozniak, 2012), work from several laboratories was described showing that HSV1 infection of cultured cells, including human neural cells, causes a marked accumulation of intracellular Aβ and P-tau. Also, Aβ deposits are formed in the brains of HSV1-infected mice (Wozniak et al., 2007). These increases were explained by increases in the relevant enzymes which, in the case of BACE, occur by HSV1-induced PKR activation and consequent phosphorylation of eIF2α (Ill-Raga et al., 2011). Further, infected primary neuronal and astrocyte cultures show AD-like caspase-3 activation and tau cleavage (Lerchundi et al., 2011).

De Chiara et al. (2010) examined the effects of HSV1 infection on processing of APP in human neuroblastoma cells and rat cortical neurons. They found that HSV1 caused multiple types of cleavage of APP, some of which were produced through the action of α- and β-secretases and caspase-3-like enzymes—processes that occur in the amyloidogenic pathway—leading to the formation of several potentially neurotoxic fragments. They stressed that as their cells were not transfected to over-express APP, their data might well more closely simulate events occurring *in vivo*, and they speculated that on repeated reactivation of the virus in brain, such fragments might play a major role in the pathogenesis of AD.

Another type of approach relating HSV1 to APP was pioneered by Bearer and colleagues. They studied HSV1 assembly and transport, and discovered that APP is a component of isolated HSV1 particles, about 1000 or more APP molecules being present in each particle (Satpute-Krishnan et al., 2003). Whether or not APP is present in the virus particles within cells or, instead, it joins them during the isolation procedure, is unknown. The aim of a more recent study (Cheng et al., 2011) was to investigate the latter, and also to find whether the APP affects virus intracellular transport, and the converse—whether APP transport is affected by the virus. Results obtained using GFP-tagged virus showed that APP and HSV1 co-localize and travel together within cells and that the distribution of APP is abnormal in HSV1-infected cells. In Bearer (2012), she points out that the nascent HSV1 profoundly alters cell membrane organization and anterograde transport, processes which are essential for neuronal function and survival, with particularly great impact in the case of chronic infection.

## Would antiviral treatment help AD patients?

The merits of several antiviral agents which differ in their mechanisms of action have recently been investigated in the author’s laboratory in respect to their ability to reduce HSV1-induced P-tau and Aβ production. The most commonly used antiviral, acyclovir (ACV), which is usually administered to HSE sufferers as the pro-drug, valacyclovir (VCV), because of the much greater oral bio-availability of the latter, is very effective and very safe. ACV acts by interfering with the replication of HSV1 DNA; it targets specifically cells that contain replicating HSV1 in that its action requires phosphorylation by the viral thymidine kinase (TK), The monophosphate form of ACV is then further phosphorylated by cellular kinases to the active triphosphate form, which has a greater affinity for viral than for cellular DNA polymerase. Acyclo-GTP is incorporated into viral DNA, causing chain termination.

We argued that ACV might inhibit the HSV1-induced accumulation of Aβ and P-tau only if their formation were to depend on viral DNA replication. In fact our results (Wozniak et al., 2011) showed that ACV, and also, penciclovir and foscarnet, two other agents that inhibit the viral DNA replication, did reduce Aβ and P-tau accumulation (as well as HSV1, as expected), in HSV1-infected Vero cell cultures. However, with all three antivirals, P-tau accumulation was found to depend on HSV1 DNA replication, whereas Aβ accumulation was not. The antiviral-induced decrease in Aβ was instead attributed to the reduced number of new viruses formed, hence reducing viral spread. Also, P-tau accumulation was reducible to near zero whereas that of Aβ was reducible only to a near-normal value. In fact the lesser Aβ reduction seems desirable in view of the possibility that Aβ might function at least initially as part of the innate immune system—and there are preliminary data suggesting that it does indeed have antiviral activity (Klapper et al., in preparation)—although eventually becoming toxic when over-produced. In other words, treatment should perhaps aim not to eliminate Aβ but instead to reduce it to near-normal levels—an aim achieved by ACV.

In another study, ACV was compared with an antiviral, BAY 57–1293, which targets a different stage of viral DNA replication—the helicase-primase complex (Wozniak et al., 2013a). We found that BAY 57–1293 is more efficient than ACV in decreasing Aβ and P-tau formation as well as HSV1 replication, in infected Vero cell cultures. Also, the size of the cell clusters that form during infection were reduced much more efficiently by BAY 57–1293 than by ACV. These data suggest that BAY 57–1293 would be more effective than ACV for treating AD.

The next antiviral examined was intravenous immunoglobulin (IVIG). We reasoned that its apparently beneficial action in AD patients might result from its antiviral action rather than its anti-Aβ antibodies; also, antivirals that act on HSV1 at an infection stage before viral DNA replication might be more effective in treating AD. IVIG seemed an appropriate choice for study as it can neutralize extracellular virus and also can help (in conjunction with lymphocytes) to destroy cells acutely infected with HSV1. We found (Wozniak and Itzhaki, 2013b) that IVIG was effective at reducing accumulation of Aβ and P-tau, in infected Vero cell cultures, and that it acts synergistically with ACV, suggesting that its use in combination with ACV would be beneficial for treating AD.

Amongst the many studies on VCV action *in vivo*, one of possible relevance to future clinical trials of the antiviral for AD was conducted on rabbits latently infected with HSV1, assaying the extent of asymptomatic shedding of HSV1 DNA that was known to occur in the animals’ saliva and tears. Similar shedding in saliva and tears occurs also in humans. The effects of VCV, administered to rabbits at high doses (70–140 mg/kg), on the viral DNA in tears were examined by comparing the DNA levels in tears, collected once a day for 6 days pre-infection, and 10 days post-infection (Kumar et al., 2010). It was found that the high dose VCV treatment significantly reduced the copy number of the viral DNA.

The results of Kumar et al. point to the possibility that the progress of antiviral treatment of AD patients could be monitored by assaying viral levels in tears, and presumably in saliva also—thus using the levels as biomarkers. However, so far there has been no clinical trial of antiviral treatment for AD patients, the reason being that applications for funding have been refused by grant-giving bodies and pharmaceutical companies (the latter presumably because VCV is off-patent). Yet evidence for a role for HSV1 in the disease is steadily increasing—and every other drug tested has failed to produce the magic bullet that would target disease progression. Thus AD remains an untreatable disease.

Interestingly, VCV has been found to alleviate cognitive impairment in schizophrenia patients. A test-of-concept double-blind placebo-controlled trial by Prasad et al. (2012), assessed the effects of VCV on cognitive performance and psychopathology. Of 24 HSV1-seropositive patients aged 18–50 years, 12 were given 2 × 1.5 g VCV twice daily and 12 given placebo for 18 weeks, in addition to antipsychotics. Results showed that VCV-treated subjects improved in verbal and working memory and visual object learning, compared with the placebo group. The authors pointed out that although there is no proven causal association of HSV1 with schizophrenia, their data indicate an association of the virus with cognitive impairments, especially in schizophrenia. This study, together with those referred to above by Strandberg et al. (2003, 2005), Katan et al. (2013), and Tarter et al. (2014), that indicate that viral burden, and especially HSV1, is associated with decreased cognition, provide ammunition for the concept that HSV1 is indeed responsible for cognitive decline in some AD patients.

Very intriguingly, memantine, an *N*-methyl-D-aspartate (NMDA) receptor antagonist used for treating AD, either on its own or combined with acetylcholinesterase, has recently been shown to have strong antiviral activity. The efficacy of memantine in AD, as detailed in individual clinical trials, and meta-analyses, was recently reviewed by Rive et al. (2013). They found that it was particularly important in reducing agitation and aggression, features which are very common and are usually associated with early institutionalization and with causing especially great stress to care-givers. The authors therefore concluded that memantine provides a valuable treatment for AD. The antiviral action of memantine, which was found not to involve NMDA receptors, was discovered by Brison et al. (2014): the drug reduced the effects in mice of infection with a human neurovirulent coronavirus which causes encephalitis, and a mutant of which induces a paralytic disease involving glutamate excitotoxicity. Brison et al. found that memantine acted by interfering with replication of the virus, thereby resembling other compounds derived from adamantane that inhibit virus replication, such as amantidine, and tromantadine, an anti-HSV antiviral. They therefore proposed the use of memantine to treat certain neurological diseases of possible viral cause, such as AD.

## Conclusions

The evidence presented here for the presence of HSV1 in the elderly human brain, for its reactivation there, its interference with cellular processes and its harmful effects on cognition, is very strong. The evidence for its absence, or its lack of association with APOE-ε4 in AD, was presented in three publications—10, 12 years and 15 years ago. There is therefore very good support for HSV1 being a causal factor in AD. However, with human diseases, for which cell or animal studies cannot provide wholly relevant information, and for which no direct experiments can be carried out *in vivo*, the only absolute proof of causation would be successful prevention of the disease by vaccination against the virus, or reduction of disease progression by antiviral treatment. Vaccination would have to be given in early infancy because of the very young age of primary infection, and those vaccinated would have to be followed for six or seven decades thereafter—an unrealistic possibility. This leaves antiviral treatment as a desirable, safe and potentially effective alternative.

## Conflict of interest statement

The author declares that the research was conducted in the absence of any commercial or financial relationships that could be construed as a potential conflict of interest.

## Footnotes

^1^HSV is the abbreviation used here when the method for detecting antibodies was not specific for HSV1 or HSV2. However, it is very likely indeed that the virus detected was in each case HSV1, as a far higher proportion of the elderly have been infected with this virus compared to HSV2, and also, HSV2 encephalitis is extremely rare in adults.
